# Genome Sequence of *Bacillus pumilus* MTCC B6033

**DOI:** 10.1128/genomeA.00327-14

**Published:** 2014-04-17

**Authors:** Jacylyn Villanueva, Jack Switala, Anabella Ivancich, Peter C. Loewen

**Affiliations:** a Department of Microbiology, University of Manitoba, Winnipeg, MB, Canada; b CNRS, Unité de Recherche Mixte CNRS/CEA/Université Paris-Sud (UMR 8221), Laboratoire de Bioénergétique, Métalloprotéines et Stress, Centre d’Etudes de Saclay, iBiTec-S, Gif-sur-Yvette, France

## Abstract

*Bacillus pumilus* is a Gram-positive, rod-shaped, aerobic bacterium isolated from the soil. *B. pumilus* strain B6033 was originally selected as a biocatalyst for the stereospecific oxidation of β-lactams. Here, we present a 3.8-Mb assembly of its genome, which is the second fully assembled genome of a *B. pumilus* strain.

## GENOME ANNOUNCEMENT

*Bacillus pumilus* is a soil bacterium with cellular features similar to those of other members of the *Bacillus* genus. Various attributes of the species have been exploited industrially, and *B. pumilus* strain B6033, originally isolated in India, was selected in a screen for a biocatalyst to effect the stereospecific oxidation of β-lactams to their (R)-sulfoxide derivatives (1). Subsequently, the enzyme responsible for the oxidation was isolated, and based on its dual capability to react as a catalase (H_2_O_2_ disproportionation) and a peroxidase (oxidation of a typical substrate), it was concluded to be a catalase-peroxidase (KatG) but with physical properties somewhat different from those of all other characterized KatGs (1). The putative existence of such an unusual KatG was of interest because it had the potential to shed light on the evolution and *in vivo* role of an extensively studied class of relatively new enzymes, but one which still presented many questions (2). KatGs are best known for their role in the activation of isoniazid as an antituberculosis drug, wherein mutations in the *katG* gene give rise to isoniazid resistance in *Mycobacterium tuberculosis* (3). KatGs are phylogenetically and structurally linked to the peroxidase family (4, 5), but their catalatic activity is predominant by several orders of magnitude. A side-by-side comparison has revealed a family with remarkably similar properties, making the enzyme from *B. pumilus* an apparent outlier and therefore interesting in its own right (6). In order to produce the large quantities of protein needed for a complete characterization, we wanted to clone the putative *katG* gene, and therefore, we set out to determine the sequence of the genome.

The genome of *B. pumilus* B6033 was sequenced in two stages. The first stage employed data generated using an Illumina MiSeq platform, which was assembled into 14 contigs using a combination of MIRA Assembler version 3.9.3 (7), Velvet version 1.2.08 (8), the MUMmer version 3.23 (9) package, and some Sanger sequencing. The second stage to complete the genome utilized a Pacific Biosciences data set generated by Genome Québec, which was assembled using the PacBio SMRT Analysis pipeline version 2.0.1, with 172× coverage to give a single contiguous genome sequence. The 14 contigs from the Illumina data were aligned for confirmation. The sequence was annotated by the National Center for Biotechnology Information (NCBI) Prokaryotic Genome Annotation Pipeline.

The genome sequence of *B. pumilus* MTCC B6033 consists of 3,763,493 bases, with a G+C content of 41.4%. There are 3,659 putative coding sequences, 81 tRNA genes, and 6 rRNA clusters. A comparison of the genome with the only other completed *B. pumilus* genome, that of strain SAFR-032 (accession no. NC_009848.1) (10), using Mauve 2.3.1 (11) revealed 92% identity, with rearrangements of only two small sections. Relevant to the initial purpose of the work, a catalase-peroxidase gene was not found. However, two functional catalase genes were found, one for a typical clade 1 monofunctional heme catalase and the second for a manganese catalase, in addition to a cryptic gene for a monofunctional catalase.

### Nucleotide sequence accession number.

The genome sequence of *B. pumilus* MTCC B6033 was deposited with NCBI GenBank under the accession no. CP007436.1.
